# The Thin White Line: Adaptation Suggests a Common Neural Mechanism for Judgments of Asian and Caucasian Body Size

**DOI:** 10.3389/fpsyg.2019.02532

**Published:** 2019-11-15

**Authors:** Lewis Gould-Fensom, Chrystalle B. Y. Tan, Kevin R. Brooks, Jonathan Mond, Richard J. Stevenson, Ian D. Stephen

**Affiliations:** ^1^Department of Psychology, Macquarie University, Sydney, NSW, Australia; ^2^Department of Psychology, University of Melbourne, Melbourne, VIC, Australia; ^3^Faculty of Medicine and Health Sciences, Universiti Malaysia Sabah, Kota Kinabalu, Malaysia; ^4^Perception in Action Research Centre (PARC), Macquarie University, Sydney, NSW, Australia; ^5^Body Image and Ingestion Group, Faculty of Human Sciences, Macquarie University, Sydney, NSW, Australia; ^6^Centre for Rural Health, University of Tasmania, Launceston, TAS, Australia; ^7^School of Medicine, Western Sydney University, Sydney, NSW, Australia

**Keywords:** body perception, visual adaptation, visual aftereffects, cross-cultural, body image, body size

## Abstract

Visual adaptation has been proposed as a mechanism linking viewing images of thin women’s bodies with body size and shape misperception (BSSM). Non-Caucasian populations appear less susceptible to BSSM, possibly because adaptation to thin Caucasian bodies in Western media may not fully transfer to own-race bodies. Experiment 1 used a cross-adaptation paradigm to examine the transfer of body size aftereffects across races. Large aftereffects were found in the predicted directions for all conditions. The strength of aftereffects was statistically equivalent when the race of test stimuli was congruent vs. incongruent with the race of adaptation stimuli, suggesting complete transfer of aftereffects across races. Experiment 2 used a contingent-adaptation paradigm, finding that simultaneous adaptation to wide Asian and narrow Caucasian women’s bodies (or vice versa) results in no significant aftereffects for either congruent or incongruent conditions and statistically equivalent results for each. Equal and opposite adaptation effects may therefore transfer completely across races, canceling each other out. This suggests that body size is encoded by a race-general neural mechanism. Unexpectedly, Asian observers showed reduced body size aftereffects compared to Caucasian observers, regardless of the race of stimulus bodies, perhaps helping to explain why Asian populations appear less susceptible to BSSM.

## Introduction

Body size and shape misperception (BSSM) – a phenomenon in which some individuals overestimate or underestimate their body size – affects a large and growing segment of the population of Western countries (Powell et al., 2010; Quick et al., 2015) and is becoming increasingly common in Asian societies and around the world (Wardle et al., 2006). Body size overestimation is associated with high levels of body dissatisfaction, anxiety, and depression and is a risk factor for the development of eating disorders and compulsive exercise behaviour, particularly in young women (Griffiths et al., 2016; Bould et al., 2018). In Malaysia, a rapidly developing Southeast Asian country, 20.5% of underweight young women in a large epidemiological study overestimated their body size (Khor et al., 2009), while a large longitudinal study of young people in 24 Western countries found that over 40% of non-overweight young women overestimated their body size (Quick et al., 2015). Meanwhile, approximately a quarter of overweight (BMI > 25) women and half of overweight men misperceive themselves as having the “right weight” (Yaemsiri et al., 2011), suggesting they may be underestimating their body size. This form of BSSM may be associated with reduced motivation to lose weight (Powell et al., 2010).

Explanations for body size overestimation have typically focused on the role of the Western media’s portrayal of idealized thin female bodies (Groesz et al., 2002). This connection between exposure to Western media and body image distortion is well established, with recent studies showing that the introduction of television into a region is associated with thinner ideal body size (Boothroyd et al., 2016), even when controlling for potential confounds such as nutritional status (Jucker et al., 2017). However, some evidence suggests that exposure to idealized bodies may have less pronounced effects on body dissatisfaction (which is affected by BSSM; Bould et al., 2018) for individuals of races not represented in the images (DeBraganza and Hausenblas, 2010).

Explanations for how exposure to idealized media portrayals of thin bodies leads to body image distortion have tended to focus on sociocultural mechanisms such as social comparison. However, there is evidence that mere exposure to a visual diet containing these images is sufficient to induce changes in body size preferences (Stephen and Perera, 2014) independent of cues to the social status of the individuals depicted (Boothroyd et al., 2012). Similarly, it has been suggested that body size underestimation by obese individuals may be attributable to a visual diet in which overweight and obese bodies are common, as overweight and obesity become more common in the population (Robinson and Kirkham, 2014).

Recently, it has been suggested that visual adaptation may offer a mechanism for the development of both overestimation and underestimation of body size (Mohr et al., 2016; Challinor et al., 2017; Sturman et al., 2017). Visual adaptation is a phenomenon whereby prolonged exposure to a particular stimulus (e.g., downward motion) causes adaptation of the associated neurons such that subsequently presented stimuli (stationary objects) appear to have properties opposite to those shown by the adapted stimulus (to be moving upward). These visual adaptation effects are observed in a range of low-level properties of stimuli, including orientation, color, depth, and spatial frequency (Thompson and Burr, 2009). More recently, visual adaptation has been demonstrated in higher level stimulus properties, including the identity (Rhodes et al., 2011) and gender (Yang et al., 2011) of faces, and the size and shape of bodies (Mohr et al., 2016; Sturman et al., 2017). For bodies, exposure to wide (narrow) figures causes a change in perceived size such that subsequently viewed body images appear narrower (wider) than they are (Challinor et al., 2017). Hence, the point of subjective normality (PSN) moves toward the adaptation stimulus.

For faces, the extent of visual adaptation depends in part on the race of the stimuli. Participants exposed to distorted images of faces of one race (e.g., expanded Asian faces) show an aftereffect of perceived contraction that is larger when assessed using test faces of the same race than for test faces of a different race (e.g., Caucasian faces) – a phenomenon known as partial cross-adaptation (Jaquet et al., 2008). Further, when exposed to faces of different races distorted in opposite directions (e.g., expanded Asian and contracted Caucasian faces), participants show “contingent adaptation” – simultaneous visual adaptation in both races in opposite directions (e.g., subsequently presented Asian faces appeared contracted, while Caucasian faces appeared expanded) (Jaquet et al., 2008; Gwinn and Brooks, 2013; Gwinn and Brooks, 2015a). This suggests that faces of different races are encoded by partially dissociated sets of neurons. Similar dissociations have also been demonstrated for the encoding of faces of different genders and ages (Yang et al., 2011).

Partial transfer of body size aftereffects and category-contingent body size adaptation have also been demonstrated. Brooks et al. (2016) exposed participants to adaptation stimuli with a particular identity (e.g., wide other-identity bodies), showing aftereffects of reduced magnitude when tested using stimuli with identities different to the adaptation stimuli (e.g., their own body). Furthermore, participants exposed to different-identity bodies that have been distorted in opposite directions (e.g., wide own-identity and narrow other-identity bodies) show simultaneous aftereffects in opposite directions in each body identity (e.g., own-identity bodies appear narrower and other-identity bodies appear wider than they are). This suggests that own- and other-identity bodies are encoded by partially dissociated neural populations (Brooks et al., 2016). It is not known, however, whether bodies of different races are encoded using independent or partially dissociated neural populations or a single population that is not race-sensitive. If there were no transfer of body size aftereffects, or if transfer was only partial, it may provide an explanation for the reduced effects of viewing other- (compared to own-)race bodies on observers’ body dissatisfaction (DeBraganza and Hausenblas, 2010) and may shed light on the observed lower rates of body image disturbance in cultures where western media promotes the thin ideal using Caucasian models to populations of other races. For example, exposure to mainstream television (which typically features primarily white bodies) had no effect on African American women’s body image, whereas exposure to black-oriented media (which typically includes black bodies and larger body sizes) improved their body image (Schooler et al., 2004).

Here, we present two experiments that aim to determine the extent of transfer of body size aftereffects across bodies of different races and thus to shed light on the nature of the neural populations encoding body size in bodies of different races. Based on the known dissociation of neural populations for the processing of facial distortion, we hypothesized that participants would show partial transfer of body size adaptation effects between bodies of different races.

## Methods

All work was approved by both the Macquarie University Human Research Ethics Committee (MUHREC), as well as the Medical Ethics Committee of the Universiti Malaysia Sabah (MECUMS). All participants gave prior, informed consent in writing.

### Experiment 1

Experiment 1 used a cross-adaptation paradigm to elucidate whether the neural populations encoding body size for Asian and Caucasian identities are common or independent. Participants were exposed either to Asian or Caucasian adaptation stimuli and tested with identities from both races. If independent race-specific neural populations are used for the encoding of body size for each race, we predict that aftereffects should be demonstrated when test bodies are from the same racial group as the adaptation stimuli but absent when the adaptation and test stimuli are of different races (i.e., zero transfer of the aftereffect). Alternatively, encoding of body size by a common, race-general population would predict equivalent aftereffect magnitudes for Asian and Caucasian test identities regardless of the race of adaptation stimuli (i.e., 100% transfer). An intermediate arrangement is also possible, such that that neural populations show partial overlap. This allows us to predict aftereffects for all test bodies, but that these should be stronger for Asian compared to Caucasian test identities following adaptation to Asian adaptation identities, and vice versa (partial transfer).

#### Methods

##### Participants

One hundred and twenty-seven female participants were recruited in Australia (*n* = 67) and Malaysia (*n* = 60), giving 95% power to detect a small-medium effect size for the hypothesized race of adaptor × race of test stimulus × direction of adaptation interaction in the planned ANOVA (calculated using G*Power v3.1.9.2). Australia-based participants (*M*_age_ = 21.8, SD = 5.23), self-identified as Caucasian, were primarily first-year undergraduates and received course credit for their participation. Malaysia-based participants (*M*_age_ = 24.6, SD = 5.72), who self-identified as East/Southeast Asian, were a mix of family and friends of the researcher and students at the Universiti Malaysia Sabah and were entered into a draw to win one of three RM50 gift cards for their participation. Two Australian and four Malaysian participants were excluded from the final analysis because they did not identify as “Caucasian” (in Australia) or “East/South East Asian” (in Malaysia). Additionally, five Australian participants were excluded from the final analysis because of technical problems during their participation sessions (software crashing midway through the testing session), leaving a total of 116 participants.

##### Stimuli

Color, full-body photographs of Caucasian and Asian individuals were obtained from a database built for a larger study. Photographs were taken with participants wearing standard gray, tight-fitting shorts, and singlet, standing in a Munsell N5 gray-painted photo booth, under standardized lighting and with all camera settings held constant (Sturman et al., 2017).

**Figure 1:**
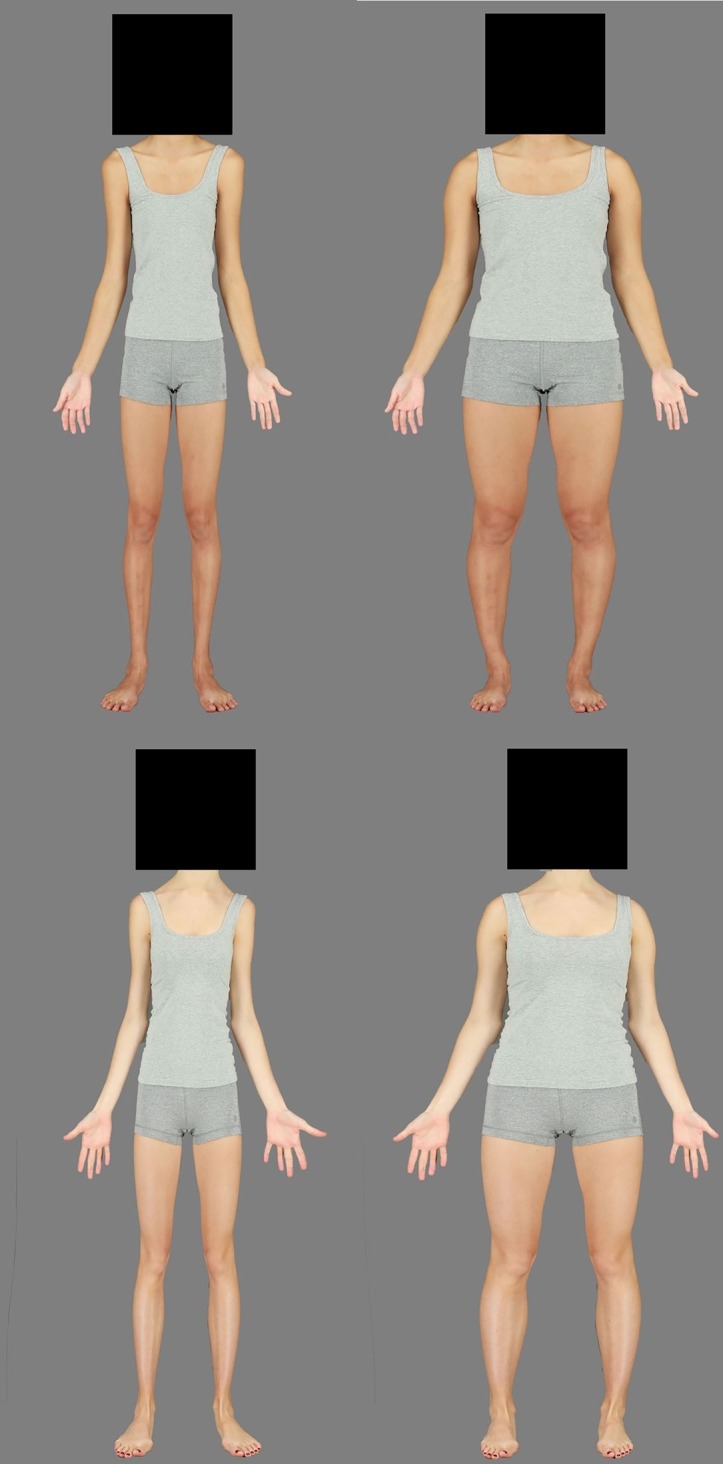
Examples of Asian (top) and Caucasian (bottom), contracted (left) and expanded (right) adaptation stimuli, matched on initial BMI. All faces were visible during experimentation.

Twenty-two Asian and 22 Caucasian identities were used in this study. Height and weight measurements were taken and BMI (Asian *M* = 22.19, SD = 3.14; Caucasian *M* = 22.18, SD = 3.22) calculated, which were then used to match each Asian identity to a corresponding Caucasian identity. Images were resized to 450 × 900 pixels, and all identifying markings or tattoos were digitally removed using the clone stamp tool in Adobe Photoshop CS6. All identities used were within one SD of the mean BMI in the larger database. For each identity, the body (from the neck downward) was expanded horizontally by 50% and contracted by 50%, using the Spherize tool in Photoshop (which has previously been shown to produce stimuli that successfully induce body size adaptation effects; Brooks et al., 2016). This created two images of the same identity that appeared to differ drastically in body fat. These were used as the adaptation stimuli (Figure 1).

**Figure 2:**
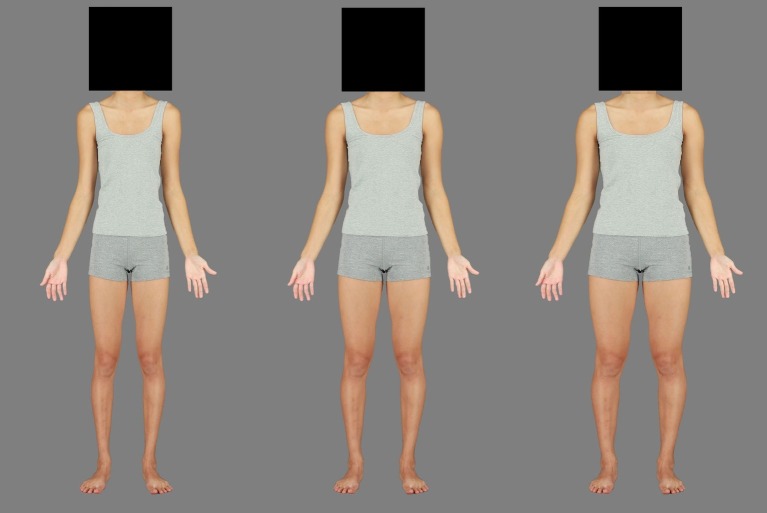
Original (center) and contracted (left) and expanded (right) endpoints of the manipulation used to produce the test stimuli. All faces were visible during experimentation.

Further 12 images, horizontally expanded or contracted in 5% increments, up to and including ±30%, were created as the test stimuli to add to the original “0%” image (Figure 2). The faces of each identity remained visible so that race could easily be discerned, but faces were left unaltered by the Spherize manipulation to avoid the possibility of confounding effects of face adaptation.

##### Procedure

Participants were pseudorandomly assigned to one of four adaptation conditions to determine the adaptation stimuli with which they would be presented – Expanded Asian (A+), Expanded Caucasian (C+), Contracted Asian (A−), or Contracted Caucasian (C−). Participants provided their age, race, and sex *via* a demographics questionnaire in Qualtrics, before proceeding to the practice phase. The practice phase consisted of a series of two Asian and two Caucasian test identities (each Asian test identity matched for initial BMI with a Caucasian test identity), presented once each, one at a time in random order. A method of adjustment task programmed in Matlab R2017b with Psychtoolbox 3 was used. For each test identity, moving the mouse horizontally cycled through the 13 frames of the test identity’s transformation. Participants were instructed that moving the mouse horizontally would change the appearance of the body (but were not told it was a width manipulation) and were asked to “make the body look as normal as possible”, then click to save the data, and move onto the next identity. The initial frame presented was randomized. Each trial was preceded by a beep to indicate that a response was required.

Next, in the baseline test phase, participants were presented with a series of 20 test identities (10 Asian and 10 Caucasian, each Asian identity matched for initial BMI with a Caucasian identity), selected at random from the pool, once each, one at a time in random order, and asked to make them appear as normal as possible.

In the adaptation test phase, participants were presented with a 2-min slide show of 10 identities according to their condition (expanded or contracted Asian or Caucasian bodies). Each identity was presented six times, for a total duration of 12 s. Participants were not required to make a response, and no beep sound was played. Following this initial adaptation, participants were presented with the same 20 test identities from the baseline phase (10 Asian and 10 Caucasian, matched on initial BMI), once each, one at a time, in random order, and asked to make the bodies as normal as possible. Between each trial, a series of six “top up” adaptation stimuli were selected at random from the adaptation identities and presented for 1 s each (a total of 6 s) to ensure the maintenance of the adaptation effect (Brooks et al., 2016). Participants were asked to simply observe the top up stimuli without making a response.

##### Data Processing and Analysis

Baseline point of subjective normality (PSN) was calculated for each participant as the average amount of expansion/contraction chosen to make the bodies look as normal as possible across the baseline trials. Adapted PSN was calculated for each participant as the average amount of expansion/contraction chosen across the adaptation test phase trials. Change in PSN (ΔPSN) was calculated by subtracting baseline PSN from adapted PSN, such that a positive (negative) value indicated that the participant’s perception of normal body size had become wider (narrower) as a result of adaptation. This was used as the main dependent variable.

Separate one-sample *t*-tests were used to test for the presence of aftereffects in each of the four conditions for each of the test races. A 2 (race of test stimuli; within subjects) × 2 (race of adaptation stimuli; between subjects) × 2 (race of participant; between subjects) × 2 (direction of adaptation; expanded or contracted; between subjects) mixed ANOVA was used to determine whether there was significant transfer of adaptation across bodies of different races, for participants of different races.

#### Results

**Figure 3:**
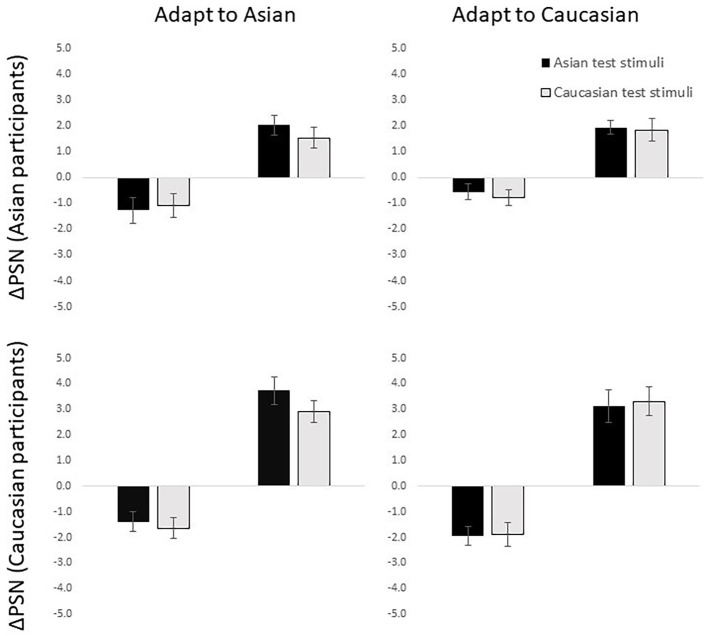
ΔPSN scores for Asian (top) and Caucasian (bottom) participants adjusting Asian (black bars) and Caucasian (light bars) test identities following exposure to Asian (left) and Caucasian (right) adaptation stimuli. Error bars show standard error of the mean.

The average ΔPSN for each condition was found to be significantly different from 0 in the predicted direction (Figure 3; those exposed to expanded/contracted adaptation stimuli showed positive/negative ΔPSN scores) across all eight one-sample *t*-tests (all *p* < 0.001), suggesting that aftereffects were successfully induced in the predicted direction in all eight conditions.

In the ANOVA, as predicted, a significant main effect of direction of adaptation was significant, *F*(1,108) = 177.72, *p* < 0.001, $\eta_{p}^{2}$ = 0.62, with those exposed to expanded adaptation conditions showing positive ΔPSN scores (*M* = 2.59, SD = 1.83) and those in the contracted adaptation conditions showing negative ΔPSN scores (*M* = −1.33, SD = 1.42). Unexpectedly however, there was a significant interaction between direction of adaptation and race of participant, *F*(1,108) = 14.60, *p* < 0.001, $\eta_{p}^{2}$ = 0.12. Caucasian participants appeared to be experiencing stronger average body size aftereffects compared to the Asian participants in both expanded, *t*(49.44) = −3.19, *p* = 0.002, *d* = 0.91 (Caucasian *M* = 3.27, SD = 2.01; Asian *M* = 1.84, SD = 1.26), and contracted, *t*(57) = 2.16, *p* = 0.035, *d* = 0.57 (Caucasian *M* = −1.71, SD = 1.33; Asian *M* = −0.93, SD = 1.42) adaptation conditions. No other significant main effects or interactions were found (all *p*’s > 0.05).

Importantly, there was no significant interaction found between race of test stimuli and race of adaptation stimuli, *F*(1,108) = 1.67, *p* = 0.199, $\eta_{p}^{2}$ = 0.02, suggesting that transfer of aftereffects was complete across stimulus races. The lack of a significant result in a standard null-hypothesis significance test (NHST) should not, however, be taken as evidence of no difference between groups (absence of evidence is not evidence of absence). This result was therefore followed up with equivalence testing. This technique reverses the typical assumptions of NHST, setting the null hypothesis to “there is a difference between the means,” and the alternative hypothesis to “the means are equivalent” (Lakens, 2017). The two one-sided tests (TOST) equivalence testing method was used to determine if the aftereffects were significantly equivalent in conditions where the race of the test stimuli was congruent vs. conditions where the race of the test stimuli was incongruent with the race of the adaptation stimuli. Upper and lower bounds were set to *d*_z_ = ±0.35 (equivalent to a ±2.5% spherize transform – half of the difference between two adjacent frames in the transform sequence) (Lakens, 2017). The TOST procedure indicated that the observed effect size (*d*_z_ = −0.12) was significantly within the equivalent bounds, *t*(115) = 2.65, *p* = 0.030.

#### Discussion

Experiment 1 found significant body size adaptation in the predicted directions in all conditions, such that exposure to expanded bodies resulted in an aftereffect of body size underestimation (i.e., more expanded bodies being perceived as normal), while exposure to contracted bodies resulted in body size overestimation (i.e., more contracted bodies being perceived as normal). The magnitude of the aftereffect was significantly equivalent in congruent and incongruent race conditions, suggesting that the transfer of adaptation across races of bodies was complete. Finally, Asian participants appeared to experience a significantly weaker body size adaptation effect compared to Caucasian participants, regardless of the race of the test and adaptation stimuli.

### Experiment 2

Experiment 2 used a contingent adaptation paradigm, in which participants completed a method of adjustment task to determine the body size that they perceived as most “normal” (point of subjective normality; PSN) before and after exposure to adaptation bodies, which were either expanded Asians and contracted Caucasians or contracted Asians and expanded Caucasians. Following the results of Experiment 1, we predicted that there would be complete transfer of body size aftereffects across races of bodies, with a canceling effect leading to much reduced or even absent aftereffects in both races of stimuli.

#### Methods

##### Participants

Forty-five female participants were recruited in Sydney, Australia and Kota Kinabalu, Malaysia. Twenty-four Australian Caucasian participants (*M*_age_ = 19.5, SD = 2.25) were recruited in Sydney, Australia and 21 East/Southeast Asian Malaysian participants (*M*_age_ = 24.4, SD = 2.25) were recruited in Kota Kinabalu, Malaysia in the same way as Experiment 1, giving 95% power to detect a small-medium effect size for the hypothesized test race × participant race × adaptation condition interaction in the planned ANOVA (calculated using G*Power v3.1.9.2). Two Caucasian participants were excluded from the final analysis because they responded identically to every test stimulus.

##### Stimuli

The stimuli were the same as for Experiment 1.

##### Procedure

Experiment 2 employed a 2 (test race; within-subjects) × 2 (adaptation condition; between subjects) × 2 (participant race; between subjects) mixed factorial design. Participants were pseudorandomly assigned to one of two conditions, one in which participants were exposed to five expanded Asian and five contracted Caucasian adaptation stimuli, matched for initial BMI (A+/C−) and the other in which participants were exposed to five contracted Asian and five expanded Caucasian adaptation stimuli, matched for initial BMI (A−/C+), six times each for a total of 2 min. Top up stimuli in the adapted test phase (three Asian and their initial BMI-matched Caucasian counterparts) were drawn at random from these adaptation stimuli each trial. All other aspects of the procedure were the same as Experiment 1.

##### Data Processing and Analysis

ΔPSN was calculated as in Experiment 1. Separate one-sample *t*-tests, for participants of each race, in each adaptation condition, and for test bodies of each race, were conducted comparing ΔPSN values against a reference value of 0 to determine whether PSNs changed from baseline to adapted test phase. A 2 (adaptation condition) × 2 (race of test body) × 2 (participant race) mixed ANOVA was used to examine the effects of race on the magnitude and direction of the aftereffect.

For conditions in which the adaptation and test stimuli were incongruent, scores were multiplied by −1, such that a positive result indicated an adaptation effect in the direction predicted if race-contingent adaptation was to occur. TOST was used to test for equivalence of effects between all congruent and incongruent conditions.

#### Results

No significant change in PSN was found for participants of either race, in either adaptation condition, for test bodies of either race (all *p*’s > 0.05).

In the ANOVA, no significant main effects or interactions were found (all *p*’s > 0.05), indicating that significant contingent aftereffects were not detected. The TOST procedure indicated that the observed effect size (*d*_z_ = −0.06) was significantly within the equivalent bounds of *d*_z_ = ±0.35 (±4% of spherize transform – less than the difference between two adjacent frames in the transform sequence), *t*(42) = 1.90, *p* = 0.032, suggesting that aftereffects were of equivalent size in congruent and incongruent conditions.

#### Discussion

Participants did not show any significant change in their perception of normal body size after adaptation to stimuli of different races whose size had been manipulated in opposite directions. This result has a number of possible interpretations. Firstly, it is possible that no meaningful visual adaptation took place. However, given that this experiment was modeled on a previous investigation that showed significant contingent adaptation under highly similar conditions (Brooks et al., 2016) and given the significant adaptation effects detected in Experiment 1, this seems unlikely. An alternative explanation is that the neural populations processing body size for Asian and Caucasian bodies are largely or completely overlapping. In this case, the transfer of body size adaptation across races is predicted to be large or complete, as revealed by the significant equivalence test, such that the adaptation to the expanded and contracted bodies cancel each other out, to result in no net effect. This interpretation is consistent with the finding of no reduction in visual body size aftereffects for race-incongruent conditions in Experiment 1.

## General Discussion

Across both experiments, participants showed equivalence in their adaptation to bodies of different races. In Experiment 1, when exposed to only expanded or contracted versions of only one race during the adaptation phase, participants exhibited body size adaptation effects in the same direction and of equivalent magnitude in test stimuli of both races. It was noted, however, that Asian participants exhibited body size adaptation effects of reduced magnitude compared to Caucasian participants. In Experiment 2, participants showed no overall body size aftereffects when exposed to expanded Asian and contracted Caucasian adaptation bodies (or vice versa) in the same session, and aftereffects were equivalent for congruent and incongruent pairings of race of adaptation stimuli and race of test stimuli implying that, rather than resulting in simultaneous adaptation in opposite directions in different races, this exposure led to a cancellation effect.

A number of studies have demonstrated contingent adaptation for different race faces (Jaquet et al., 2008; Gwinn and Brooks, 2013; Gwinn and Brooks, 2015a,b) and partial transfer of visual adaptation across faces of those same races (Jaquet et al., 2008), with authors inferring that the processing of faces of different races takes place in partially dissociated neural populations. Similarly, partial transfer of body size aftereffects has been demonstrated across bodies of different identities (own- vs. other-identity bodies), suggesting partial dissociation of the neural populations encoding body width in one’s own vs. other individuals’ bodies (Brooks et al., 2016). Similar results have been shown for different gendered bodies (Brooks et al., 2019). However, our results suggest that body size aftereffects show complete transfer across bodies of different races, suggesting that a single, race-general neural population encodes the width of bodies of different races.

Contingent adaptation is dependent upon the classification of target stimuli into different groups (Little et al., 2008). The lack of race-contingent body size adaptation effects in the current studies may therefore reflect a lack of automatic categorization of bodies by race. This lack of racial categorization based on bodies is also reflected in studies examining the other-race effect (the well-known phenomenon in which it is more difficult to recognize individuals of a different race than individuals of one’s own race). While the other-race effect is well established in faces (Hayden et al., 2007), similar effects can be induced in own-race faces by the induction of outgroup classification of faces, suggesting that outgroup classification may be necessary to induce the other-race effect (Zheng and Segalowitz, 2014). The other-race effect appears to be absent in bodies, however, providing further support for the suggestion that racial classification may not be automatic in bodies (Humphreys et al., 2005).

Unexpectedly, we found that Malaysian participants exhibited reduced body size adaptation effects compared to Australian participants across test and adaptation stimuli of both races. Further research is required to explain this phenomenon. However, we note that this novel finding fits with a broader pattern of differences between Caucasian and Asian participants in their performance on visual cognition tasks, including differences in the processing of faces (Blais et al., 2008), which some authors have attributed to broader cultural differences in cognitive styles (Blais et al., 2008). Our results suggest that racial/cultural differences in visual processing result in reduced susceptibility to visual adaptation among Asian populations. Although it is impossible to distinguish the environmental from the biological origins of these perceptual differences, it may be productive to investigate cultural differences in cognitive styles as an explanation for the origins of this difference in susceptibility to body size adaptation effects. One possible mechanism by which this may operate is that Asian participants may have directed more of their visual attention to the faces, and less to the bodies, than Caucasian participants, perhaps due to stronger cultural taboos against looking at bodies. However, it should be noted that Australia and Malaysia also differ in their degree of economic development, with Australia having an estimated GDP per capita of US$50,400 (CIA, 2018), compared to Malaysia’s $29,100 (CIA, 2017), and economic development has been previously linked to BMI preferences (Furnham et al., 2007), possibly *via* increased exposure to media representations of thin idealized bodies as economic development increases (Boothroyd et al., 2016).

The body size aftereffect has been demonstrated to manipulate body misperception and dissatisfaction in quite negative ways across Western populations. Due to the pervasion of thin media images in modern Western, and increasingly Asian, society, understanding why individuals in certain cultures are less susceptible to this effect may have implications for interventions designed to reduce the harm associated with BSSM.

In conclusion, our results do not support the suggestion that the reduced body dissatisfaction seen in some non-Caucasian groups (DeBraganza and Hausenblas, 2010) can be explained by partial transfer of body size aftereffects from thin-idealized Caucasian bodies in the media to their own bodies. However, the unexpected demonstration of smaller body size aftereffects for Asian participants in general offers promise in providing a potential explanation for the reduced levels of body dissatisfaction shown in these cultures.

## Data Availability Statement

The raw data supporting the conclusions of this manuscript will be made available by the authors, to any qualified researcher.

## Ethics Statement

The studies involving human participants were reviewed and approved by Macquarie University Human Research Ethics Committee and the Medical Ethics Committee of the Universiti Malaysia Sabah. The patients/participants provided their written informed consent to participate in this study.

## Author Contributions

LG-F and IS helped in conception, design, collection, analysis, and interpretation of data and contributed in drafting the article. CT Collected data and helped in revision of the article. KB helped in conception, design, interpretation of data, and revision of article. JM contributed in design, interpretation of data, and revision of the article. RS helped in interpretation of data and revision of the article.

### Conflict of Interest

The authors declare that the research was conducted in the absence of any commercial or financial relationships that could be construed as a potential conflict of interest.

## References

[ref1] Blais C. Jack R. E. Scheepers C. Fiset D. Caldara R. (2008). Culture shapes how we look at faces. PLoS One 3:e3022. 10.1371/journal.pone.0003022 18714387 PMC2515341

[ref2] Boothroyd L. G. Jucker J. L. Thornborrow T. Jamieson M. A. Burt D. M. Barton R. A. (2016). Television exposure predicts body size ideals in rural Nicaragua. Br. J. Psychol. 107, 752–767. 10.1111/bjop.12184 26910312

[ref3] Boothroyd L. G. Tovée M. J. Pollet T. V. (2012). Visual diet versus associative learning as mechanisms of change in body size preferences. PLoS One 7:e48691. 10.1371/journal.pone.0048691 23144929 PMC3492445

[ref4] Bould H. Carnegie R. Allward H. Bacon E. Sapseid M. Button K. (2018). Effects of visual adaptation on perception of and satisfaction with own body size: two randomised studies. R. Soc. Open Sci. 5:171387. 10.1016/S0924-977X(16)70056-3 29892352 PMC5990741

[ref500] Brooks K. R. Baldry E. Mond J. Stevenson R. J. Mitchison D. Stephen I. D. (2019). Gender and the body size aftereffect: Implications for neural processing. Front. Neurosci. 13:1100. 10.3389/fnins.2019.01100 31680834 PMC6813220

[ref5] Brooks K. R. Mond J. M. Stevenson R. J. Stephen I. D. (2016). Body image distortion and exposure to extreme body types: contingent adaptation and cross adaptation for self and other. Front. Neurosci. 10:334. 10.3389/fnins.2016.00334 27471447 PMC4946181

[ref6] Challinor K. L. Mond J. Stephen I. D. Mitchison D. Stevenson R. J. Hay P. (2017). Body size & shape misperception and visual adaptation: an overview of an emerging research paradigm. J. Int. Med. Res. 45, 2001–2008. 10.1177/0300060517726440 29076380 PMC5805224

[ref7] CIA (2017). Malaysia. World Factb. Available at: https://www.cia.gov/library/publications/the-world-factbook/geos/my.html (Accessed November 27, 2018).

[ref8] CIA (2018). Australia. CIA World Factb. Available at: https://www.cia.gov/library/publications/the-world-factbook/geos/as.html (Accessed January 24, 2018).

[ref9] DeBraganza N. Hausenblas H. A. (2010). Media exposure of the on ideal physique women’s body dissatisfaction and mood. J. Black Stud. 40, 700–716. 10.1177/0021934708317723

[ref10] Furnham A. Swami V. Tovee M. J. Furnham A. Swami V. (2007). “Healthy body equals beautiful body? Changing perceptions of health and attractiveness with shifting socioeconomic status” in The body beautiful. eds. Swami V. Furnham A. (London: Palgrave Macmillan), 108–128.

[ref11] Griffiths S. Hay P. Mitchison D. Mond J. M. McLean S. A. Rodgers B. (2016). Sex differences in the relationships between body dissatisfaction, quality of life and psychological distress. Aust. N. Z. J. Public Health 40, 508–522. 10.1111/1753-6405.12538 27372301

[ref12] Groesz L. M. Levine M. P. Murnen S. K. (2002). The effect of experimental presentation of thin media images on body satisfaction: a meta-analytic review. Int. J. Eat. Disord. 31, 1–16. 10.1002/eat.10005 11835293

[ref13] Gwinn O. S. Brooks K. R. (2013). Race-contingent face aftereffects: a result of perceived racial typicality, not categorization. J. Vis. 13, 1–11. 10.1167/13.11.13 23970436

[ref14] Gwinn O. S. Brooks K. R. (2015a). Face encoding is not categorical: consistent evidence across multiple types of contingent aftereffects. Vis. Cogn. 6285, 1–27. 10.1080/13506285.2015.1091800

[ref140] Gwinn O. S. Brooks K. R. (2015b). No role for lightness in the encoding of Black and White: Race-contingent face aftereffects depend on facial morphology, not facial luminance. Vis. Cogn. 23, 597–611. 10.1080/13506285.2015.1061085

[ref15] Hayden A. Bhatt R. S. Joseph J. E. Tanaka J. W. (2007). The other-race effect in infancy: evidence using a morphing technique. Infancy 12, 95–104. 10.1080/15250000701298923 33412731

[ref16] Humphreys G. W. Hodsoll J. Campbell C. (2005). Attending but not seeing: the “other race” effect in face and person perception studied through change blindness. Vis. Cogn. 12, 249–262. 10.1080/13506280444000148

[ref17] Jaquet E. Rhodes G. Hayward W. G. (2008). Race-contingent aftereffects suggest distinct perceptual norms for different race faces. Vis. Cogn. 16, 734–753. 10.1080/13506280701350647

[ref18] Jucker J. L. Thornborrow T. Beierholm U. Burt D. M. Barton R. A. Evans E. H. (2017). Nutritional status and the influence of TV consumption on female body size ideals in populations recently exposed to the media. Sci. Rep. 7, 1–9. 10.1038/s41598-017-08653-z 28814743 PMC5559456

[ref19] Khor G. L. Zalilah M. S. Phan Y. Y. Ang M. Maznah B. Norimah A. K. (2009). Perceptions of body image among Malaysian male and female adolescents perceptions of body image among Malaysian male and female adolescents. Singap. Med. J. 50, 303–311. http://europepmc.org/abstract/MED/19352576, PMID: 19352576

[ref20] Lakens D. (2017). Equivalence tests: a practical primer for t tests, correlations, and meta-analyses. Soc. Psychol. Personal. Sci. 8, 355–362. 10.1177/1948550617697177 28736600 PMC5502906

[ref21] Little A. C. DeBruine L. M. Jones B. C. Waitt C. (2008). Category contingent aftereffects for faces of different races, ages and species. Cognition 106, 1537–1547. 10.1016/j.cognition.2007.06.008 17707364

[ref22] Mohr H. M. Rickmeyer C. Hummel D. Ernst M. Grabhorn R. (2016). Altered visual adaptation to body shape in eating disorders: implications for body image distortion. Perception 45, 725–738. 10.1177/0301006616633385 26921409

[ref23] Powell T. M. de Lemos J. A. Banks K. Ayers C. R. Rohatgi A. A. Khera A. (2010). Body size misperception: a novel determinent in the obesity epidemic. Arch. Intern. Med. 170, 1695–1697. 10.1001/archinternmed.2010.314 20937931 PMC4874507

[ref24] Quick V. Nansel T. R. Liu D. Lipsky L. M. Due P. Iannotti R. J. (2015). Body size perception and weight control in youth: 9-year international trends from 24 countries. Int. J. Obes. 38, 988–994. 10.1038/ijo.2014.62.Body PMC4090285 24722544

[ref25] Rhodes G. Jeffery L. Evangelista E. Ewing L. Peters M. Taylor L. (2011). Enhanced attention amplifies face adaptation. Vis. Res. 51, 1811–1819. 10.1016/j.visres.2011.06.008 21704059

[ref26] Robinson E. Kirkham T. C. (2014). Is he a healthy weight? Exposure to obesity changes perception of the weight status of others. Int. J. Obes. 38, 663–667. 10.1038/ijo.2013.154 23949613

[ref27] Schooler D. Ward L. M. Merriwether A. Caruthers A. (2004). Who’s that girl: television’s role in the body image development of young white and black women. Psychology 28, 38–47. 10.1111/j.1471-6402.2004.00121.x

[ref28] Stephen I. D. Perera A. T.-M. (2014). Judging the difference between attractiveness and health: does exposure to model images influence the judgments made by men and women? PLoS One 9:e86302. 10.1371/journal.pone.0086302 24466014 PMC3896486

[ref29] Sturman D. Stephen I. D. Mond J. Stevenson R. J. R. J. Brooks K. R. (2017). Independent aftereffects of fat and muscle: implications for neural encoding, body space representation, and body image disturbance. Nat. Sci. Rep. 7:40392. 10.1038/srep40392 PMC5223140 28071712

[ref30] Thompson P. Burr D. (2009). Visual aftereffects. Curr. Biol. 19, 11–14. 10.1016/j.cub.2008.10.014 19138580

[ref31] Wardle J. Haase A. M. Steptoe A. (2006). Body image and weight control in young adults: international comparisons in university students from 22 countries. Int. J. Obes. 30, 644–651. 10.1038/sj.ijo.0803050 16151414

[ref310] Yaemsiri S. Slining M. M. Agarwal S. K. (2011). Perceived weight status, overweight diagnosis, and weight control among US adults: the NHANES 2003–2008 study. Int. J. Obes. 35, 1063–1070. 10.1038/ijo.2010.229 21042327

[ref32] Yang H. Shen J. Chen J. Fang F. (2011). Face adaptation improves gender discrimination. Vis. Res. 51, 105–110. 10.1016/j.visres.2010.10.006 20937298

[ref33] Zheng X. Segalowitz S. J. (2014). Putting a face in its place: in- and out-group membership alters the N170 response. Soc. Cogn. Affect. Neurosci. 9, 961–968. 10.1093/scan/nst069 23677488 PMC4090958

